# A Retrospect of a Further Series of Intra-Abdominal Operations

**Published:** 1902-03

**Authors:** James Swain

**Affiliations:** Professor of Surgery at University College, Bristol: Assistant-Surgeon to the Bristol Royal Infirmary


### A RETROSPECT OF A FURTHER SERIES OF INTRA-ABDOMINAL OPERATIONS.

BY

James Swain, M.S., M.D. Lond., F.R.C.S. Eng.,

*Professor of Surgery at University College, Bristol: Assistant-Surgeon to the Bristol Royal Infirmary.*

---

This fourth series is continuous with the former papers,[1] and operations performed per vaginam, herniotomy, etc., have been excluded as heretofore.

Appendicitis figures largely in the list; but considering the desperate condition of some of the cases, it is interesting to note that in this and the former (third) series there are recorded thirty-five consecutive operations for appendicitis without a death—a fact which bears testimony to the improved technique in the surgical treatment of this too common affection.

The treatment of diseases of the pancreas is one of the latest developments of abdominal surgery, and the result obtained in Case 9 is a gratifying omen for the future.

The table given below is merely arranged chronologically; but it is convenient to group together operations of a similar nature, in order that any special features of individual cases may be discussed.

*A*.—**Operations for Diseases of the Ovaries and Fallopian Tubes.**—Cases 6, 12, 13, 23, 31, 32 and 45.

Clinically this group may be divided into:—

**Extra-uterine fœtation.**—Case 13.

**Fibroma of Ovary.**—Case 31.

**Adeno-Cystoma of Ovary.**—Cases 12, 23 and 32.

**Parovarian Cyst.**—Cases 6 and 12.

**Salpingo-oöphoritis and Broad Ligament Cysts.**—Case 45.

**Extra-uterine fœtation** is almost always primarily tubal, and when diagnosed in the earlier months prompt surgical treatment is called for; and should a tubal abortion occur, any

[1] *Bristol M.-Chir. J.*, 1898, xvi. 211; 1900, xviii. 103; 1901, xix. 18, 121.

| NO. | SEX AND AGE. | DISEASE. | OPERATION. | RESULT. | REMARKS. |
| --- | --- | --- | --- | --- | --- |
| 1 | F. 45 | Malignant stricture of colon (splenic flexure) ... | Colectomy ... ... ... | Recovered.. | Intestinal obstruction of twelve days' duration at time of operation. |
| 2 | F. 12 | Appendicitis ... ... | Appendicectomy ... ... | Recovered.. | Appendix adherent to ileum and right Fallopian tube. |
| 3 | F. 23 | Appendicitis ... ... | Appendicectomy ... .. | Recovered.. | Appendix adherent in sub-cæcal fossa. |
| 4 | F. 47 | Myo-fibroma uteri—malignant disease of peritoneum. | Exploratory cœliotomy ... | Recovered.. | Fibroid size of small cocoanut. Peritoneum studded with small malignant deposits. |
| 5 | M. 64 | Malignant disease of descending colon. | Colotomy (tranverse) ... | Recovered.. | Growth size of fist fixed to parietes. Retro-peritoneal glands involved. |
| 6 | F. 33 | Parovarian cyst (right) ... | Ovariotomy ... ... | Recovered.. | Cyst contained six pints watery fluid, sp. gr. 1006, faintly alkaline, slightly albuminous. |
| 7 | F. 33 | Appendicitis (gangrenous).. | Appendicectomy ... ... | Recovered.. | Operation five and a half hours after perforation. Vaginal drainage. |
| 8 | M. 12 | Appendicitis ... ... | Appendicectomy ... ... | Recovered.. | Several enlarged glands removed from meso-cæcum and mesentery. |
| 9 | M. 48 | Pancreatitis—cholæmia ... | Cholecystotomy ... ... | Recovered.. | Persistent jaundice for seven weeks before operation. Head of pancreas hard and nodular. |
| 10 | F. 32 | Appendicitis ... ... | Complete inversion of appendix. | Recovered.. | Three attacks in twelve months preceding operation. |
| 11 | M. 24 | Appendicitis ... ... | Appendicectomy ... ... | Recovered.. | Appendix about six inches long. |
| 12 | F. 37 | Ovarian adeno-cystoma (left) — parovarian cyst (right). | Ovariotomy (left)—excision of cyst (right). | Recovered .. | Six pints of yellowish-brown fluid from main cyst, containing cholesterine, sp. gr. 1013, alkaline, loaded with albumin. |
| 13 | F. 37 | Extra-uterine pregnancy ... | Removal of products of conception and uterine appendages (left). | Recovered.. | A ruptured tubal pregnancy, with fœtus of about three months. |
| 14 | F. 40 | Appendicitis ... ... | Appendicectomy ... ... | Recovered.. | Appendix perforated and partially gangrenous in centre of abscess cavity. |
| 15 | M. 49 | Appendicitis ... ... | Appendicectomy ... ... | Recovered.. | Appendix adherent along whole length to inner and posterior aspects of cæcum. |

| NO. | SEX AND AGE. | DISEASE. | OPERATION. | RESULT. | REMARKS. |
|---|---|---|---|---|---|
| 16 | F. 53 | Malignant disease of stomach. | Exploratory cœliotomy .. | Died ... | Extensive glandular involvement. Death three weeks after operation. |
| 17 | F. 29 | Cholelithiasis ... ... | Cholecystotomy ... ... | Recovered.. | Fifty-six calculi removed. |
| 18 | F. 23 | Intestinal obstruction .. | Division of peritoneal band | Recovered.. | All abdominal symptoms disappeared from time of operation, but patient died about a fortnight later from obscure cerebral symptoms (convulsions, &c.) |
| 19 | F. 57 | Cholelithiasis ... ... | Cholecystotomy ... ... | Recovered.. | One large and one small calculus removed. "Riedel's lobe" of liver present. |
| 20 | M. 61 | Appendicitis ... ... | Appendicectomy ... ... | Recovered.. | Appendix ran behind cæcum and ascending colon, the distal end being attached to parietes. |
| 21 | F. 21 | Appendicitis ... ... | Appendicectomy ... ... | Recovered.. | Ten attacks during the two years preceding operation. |
| 22 | F. 65 | ? Intestinal obstruction ... | Exploratory cœliotomy ... | Recovered.. | Symptoms of chronic obstruction entirely relieved, though no cause was found. |
| 23 | F. 48 | Ovarian adeno - cystoma (left). | Ovariotomy ... ... | Died ... | Cyst wall had commenced to slough, and peritonitis had set in before operation. |
| 24 | M. 29 | Appendicitis ... ... | Appendicectomy ... .. | Recovered.. | Three attacks during eighteen months preceding operation. |
| 25 | M. 22 | Appendicitis ... .. | Appendicectomy ... .. | Recovered.. | Appendix closely bound to cæcum. |
| 26 | M. 14 | Appendicitis .. ... | Appendicectomy ... ... | Recovered.. | Appendix adherent at distal end to pelvic brim. |
| 27 | M. 73 | Intestinal obstruction ... | Enterostomy ... ... | Recovered.. | Matting felt in right iliac fossa. General condition too bad for accurate diagnosis. |
| 28 | F. 67 | Carcinoma of pylorus ... | Exploratory cœliotomy ... | Died ... | Pylorus adherent to neighbouring structures. Secondary deposits. |
| 29 | F. 42 | Carcinoma of rectum ... | Inguinal colostomy .. | Recovered | Growth adherent to sacrum and side of pelvis. |
| 30 | M. 67 | Pancreatitis (acute) .. | Exploratory cœliotomy — enterostomy. | Died ... | Several foci of pus in pancreas. Fat embolism. |
| 31 | F. 22 | Fibroma of ovary (right) ... | Salpingo-oöphorectomy ... | Recovered | Nodular tumour of 2½ inches diameter. |
| 32 | F. 57 | Ovarian adeno - cystoma (left). | Ovariotomy ... ... ... | Recovered | 6½ pints of thick greenish-brown fluid. Collapsed cyst weighed 5¼ lbs. |
| 33 | F. 49 | Myo-fibroma uteri ... | Retro - peritoneal hystero - myomectomy. | Recovered | Tumour weighed 1¼ lbs., and had caused retention of urine. |

| NO. | SEX AND AGE. | DISEASE. | OPERATION. | RESULT. | REMARKS. |
|---|---|---|---|---|---|
| 34 | M. 21 | Appendicitis ... ... | Appendicectomy ... ... | Recovered.. | Appendix bulbous at distal end and contained small concretion. |
| 35 | M. 7 | Appendicitis (suppurative). | Cœliotomy—drainage ... | Recovered.. | One ounce of fætid pus, circumscribed by adhesions. |
| 36 | M. 20 | Appendicitis (suppurative). | Cœliotomy—drainage ... | Recovered.. | One pint of stinking pus released from pelvis. |
| 37 | F. 40 | Appendicitis (suppurative). | Cœliotomy—drainage ... | Recovered.. | Six ounces of fætid pus. Abscess cavity extended behind cæcum and ascending colon. |
| 38 | F. 45 | Tubercular peritonitis ... | Cœliotomy ... ... ... | Recovered.. | Large omental "tumour" disappeared after operation. |
| 39 | M. 64 | Carcinoma of rectum ... | Inguinal colostomy ... | Died ... | Growth firmly fixed to sacrum. Subject to Bright's disease and valvular heart mischief. |
| 40 | M. 35 | Retro-peritoneal sarcoma | Exploratory cœliotomy—drainage. | Relieved ... | Disease apparently commenced in mesenteric glands. |
| 41 | F. 14 | Appendicitis ... ... | Appendicectomy ... ... | Recovered.. | Seven attacks in two years preceding operation. |
| 42 | M. 35 | Malignant disease of ascending colon. | Colectomy—ileo-colostomy | Recovered.. | Six inches of ileum and about half the large intestine removed. |
| 43 | F. 25 | Myo-fibroma uteri .. | Pan-hystero-myomectomy | Recovered.. | "Cup and ball" myo-fibroma causing retention of urine. |
| 44 | F. 62 | Malignant stricture of œsophagus.' | Gastrostomy ... ... | Recovered.. | Patient had taken no solid food for two months before operation. |
| 45 | F. 42 | Appendicitis—broad ligament cysts. | Appendicectomy - salpingo-oöphorectomy (left)-enucleation of cysts (right). | Recovered.. | The cysts were probably of inflammatory origin and secondary to the appendicitis. |
| 46 | M. 73 | Fæcal fistula ... ... | Enterectomy-ileo-ileostomy | Recovered.. | The fistula resulted from an enterostomy preformed for intestinal obstruction. |
| 47 | M. 19 | Appendicitis ... ... | Appendicectomy ... ... | Recovered.. | Long appendix with constriction in middle and bulbous end. |
| 48 | M. 58 | Malignant stricture of œsophagus. | Gastrostomy ... ... | Died ... | Death from septic broncho-pneumonia caused by perforation of trachea by a bougie before operation. |
| 49 | F. 47 | Perforating gastric ulcer ... | Gastrorraphy ... ... | Died ... | Operation ten hours after perforation. General peritonitis. |
| 50 | M. 68 | Malignant disease of rectum | Inguinal colostomy ... | Died ... | Extensive growth fixed at sides of pelvis and to base of bladder. Died from pneumonia. |

needless delay adds greatly to the danger of the mother. Diagnosis before rupture is by no means easy; and in a former paper[1] I referred to a case of normal pregnancy associated with a myo-fibroma uteri, in which the presence of a lateral tumour in the abdomen and milk in the breasts led to the erroneous diagnosis of a tubal pregnancy. When rupture has occurred, the existence of attacks of abdominal pain and collapse in association with irregular vaginal hemorrhages warns us of the probable nature of the case and the demand for operative interference. The following example is sufficiently typical of the symptoms and necessary treatment.

CASE 13.—**Ruptured Tubal Pregnancy.**—The patient had been married twelve years, but had not been pregnant. The last menstruation ceased on November 5th. On December 6th and 7th, and again on December 26th, she was seized with severe pain in the hypogastrium. From January 9th to January 19th some irregular vaginal hemorrhage occurred. On February 1st there was another severe attack of abdominal pain and some swelling of the lower abdomen. The temperature was practically normal throughout. When I saw her on February 7th she was evidently in great pain in the lower abdomen, and complained of much vaginal and rectal tenesmus. The hypogastrium was very tender. Under chloroform a well-defined swelling (displaced uterus), like the large end of an egg, could be felt above the inner third of Poupart's ligament on the right side. Extending from this across the lower abdomen to the left iliac fossa was an ill-defined swelling (containing fœtus) reaching to a point about one-third of the distance from the symphysis pubis to the umbilicus. Per vaginam the uterus was pushed to the right and wanting in mobility, the os uteri pointing backwards. In Douglas's pouch and extending across the pelvis, but more to the left than right, was a doughy swelling, the abdominal limits of which have been described. A median incision was made between the umbilicus and pubes. On opening the peritoneum a bloody fluid escaped. Occupying the left side of the pelvis and lower abdomen was a soft swelling shut off by adherent intestines and blood-clot. On pushing the fingers into this swelling, there was a gush of liquid blood and blood-clot. A fœtus and placenta were rapidly brought to the surface—from the centre of this blood-containing cavity—together with a dilated and ruptured left Fallopian tube, which had formed the sac of the products of conception. Large Spencer Wells forceps were placed on the left uterine appendages to control the hemorrhage, and the pelvis thoroughly cleared of blood-clot by pouring in normal saline solution. The

[1] *Bristol. M.-Chir. J.*, 1901, xix 25.

left broad ligament was then tied off, and the distal portion, with the fœtus, placenta, and ruptured tube, cut away. After a final washing of the pelvis with saline solution, the abdomen was closed without drainage. Recovery was uninterrupted.

**Solid Tumours of the Ovary** are usually characterised by free mobility. They often cause an amount of pain which is quite disproportionate to the size of the tumour, and may be associated with some peritoneal effusion—both probably due to the twisting of the pedicle. In Case 31 all these symptoms were produced by a tumour (2½ inches in diamer, with an irregular surface), which was attached by a rather broad pedicle to the right broad ligament at the hilum of the ovary. It was composed of bundles of fibrous tissue running in various directions.

The occurrence of **peritonitis** in connection with an **ovarian adeno-cystoma** should always be regarded with seriousness, and operative interference should be instituted without delay. The peritonitis not infrequently depends on commencing sloughing of the cyst wall, which existed in Cases 23 and 32. Case 23 unfortunately did not present herself for operation until there was an extensive peritonitis; and the patient died with all the symptoms of septic peritonitis, though I regret that cultures of the fluid were not taken. On the other hand, Case 32, in which sloughing of the cyst wall had commenced, was operated upon before any definite symptoms of peritonitis set in, and the patient recovered. Cultures from the fluid near the sloughy wall in this case were sterile.

The **broad ligament cysts** in Case 45 were apparently of inflammatory origin, being secondary to salpingo-oöphoritis following appendicitis. This case is referred to under appendicitis.

*B.*—**Operations for Myo-fibroma of the Uterus.**—Cases 4, 33 and 43.

Of these, Case 4 did not admit of removal by reason of the co-existence of malignant disease of the peritoneum—an association which I have seen sufficiently frequently to make me think that the relationship is causal rather than accidental. Case 33 was removed by retro-peritoneal supra-vaginal hysteromyomectomy, which to my mind is the operation of choice; but the globular character of the tumour in Case 43 necessitated its removal by pan-hystero-myomectomy, the whole tumour and uterus being cut away by opening the vaginal vault from above. The difficulty of operation for the removal of myo-fibromata by the abdominal route might almost be said to be inversely proportional to the size of the tumour. Case 43 was a small tumour of the "cup-and-ball" type, which caused the most intense pain by becoming accurately moulded to the pelvic cavity and causing pressure symptoms. Such a tumour presents great difficulty in removal; for its situation on the top of the vagina prevents it from being delivered through the abdominal wound, and the filling of the pelvic cavity leaves little or no room for the fingers to tie the ovarian and uterine vessels. The case is of further interest as being a good deal below the average age at which it is usually necessary to operate for myo-fibroma uteri.

*C.*—**Operation for Tubercular Peritonitis.**—Case 38.

Cases of tubercular peritonitis are commonly mistaken for ovarian cysts; but Case 38, which was correctly diagnosed before operation, presented a tumour in the epigastrium which resembled a pancreatic cyst. The "tumour" was composed of matted intestines and a very thick great omentum, and entirely disappeared after operation.

*D.*—**Operations on the Gall-bladder.**—Cases 9, 17 and 19.

Case 9 is referred to under "operations for diseases of the pancreas." Cases 17 and 19 were cases of gallstones; but Case 17 was complicated by an obstruction of the cystic duct, and Case 19 was associated with a Riedel's lobe of the liver, which is commonly found in cases of cholelithiasis.

*E.*—**Operations for Diseases of the Pancreas.**—Cases 9 and 30.

I have given a summary of the surgical treatment of pancreatitis in this *Journal* (December, 1901), to which reference may be made. Case 9 is an excellent example of the beneficial effect of indirect drainage of the pancreatic ducts by the operation of cholecystostomy. The jaundice in these cases is produced by the pressure of the swollen pancreatic duct on the biliary papilla, and drainage of the gall-bladder saves death from cholæmia and allows of the subsidence of the inflammatory mischief in the pancreas. The diagnosis of these cases from gallstones in the common duct and from malignant disease of the head of the pancreas is not easy; but the success attending the treatment of chronic pancreatitis shows the desirability of an exploratory incision in all doubtful cases of chronic jaundice. Case 30 was one of acute pancreatitis. Such cases resemble intestinal obstruction, and are usually so acute in character that a correct diagnosis is not made until the time of operation or after. So far as I know, no case of successful operation for acute pancreatitis has been recorded.

CASE 9.—**Chronic Pancreatitis—Cholæmia.**—Six months before I saw the patient he had had attacks of gastro-duodenal catarrh, with transient jaundice. For the past six weeks he had been persistently jaundiced, no bile passing with the motions. There had been frequent rigors, with a temperature of 101°; but there had been no pain or vomiting. There had been great loss of flesh. On opening the abdomen, the gall-bladder and bile ducts were found distended. No gallstones were found. The head of the pancreas felt hard, somewhat nodular and enlarged. Cholecystostomy was performed, and large quantities of bile came through the tube. Cultures from the fluid in the gall-bladder at the time of operation showed staphylococci and two varieties of bacilli—one coliform (Dr. Symes). The patient gained weight, bile appeared in the motions, and the jaundice disappeared. The gall-bladder was drained for about a month. A fistula, however, still remains.

That these cases probably arise from an infective inflammation of the pancreatic ducts is shown by the presence of staphylococci and bacilli in Case 9, and of streptococci in Case 30.

*F.*—**Operations upon the Stomach.**—CASES 44, 48 and 49.

Frank's method of **gastrostomy,** in which a portion of the stomach is brought to the surface under a bridge of skin, was adopted in Cases 44 and 48. This operation can be performed quickly, and ensures against leakage of gastric juice. The patient can also be fed at once, and this is of great importance, as in cases of malignant disease of the œsophagus the operation is too frequently delayed until the vital power of the sufferer is at a low ebb.

*G.*—**Operations for Diseases of the Intestines.**—CASES 1, 5, 18, 27, 29, 30, 39, 42, 46 and 50.

These may be divided into—

**Enterectomy and Colectomy.**—CASES 1, 42 and 46.

**Enterostomy and Colostomy.**—CASES 5, 27, 29, 30, 39 and 50.

**Ileo-colostomy and Ileo-ileostomy.**—CASES 42 and 46.

**Division of Peritoneal Band.**—CASE 18.

**Resection of Intestine** must be performed in two stages if intestinal obstruction be present, for the resisting power of the patient is so small under these circumstances that immediate resection is attended with a very high mortality. The obstruction should be relieved by bringing the gut to the surface and inserting a Paul's tube at the first operation; and enterectomy or colectomy, with immediate union of the divided bowel, should be performed at the second operation a week or ten days later. This was successfully done in Case 1. When, however, there is no active obstruction present at the time of operation, the resection should be performed and the ends of the divided gut united at one operation. A most extensive colectomy for malignant disease without complete obstruction is the following:

CASE 42.—**Malignant Disease of Ascending Colon—Colectomy—Ileo-colostomy.**—For nine months the patient had complained of attacks of sickness, constipation and abdominal pain; and a week before I saw him a "lump" had been found on the right side of the abdomen. This "lump" was a hard irregular mass, rather larger than the fist, situated in the right iliac fossa. The swelling caused a slight bulging of the anterior parietes, moved slightly with respiration, and could be moved about one inch in an upward direction and rather less from side to side. From the position and comparative fixity of the tumour it was regarded as an affection of the ascending colon, and the fact that there was distinct though slight mobility of the swelling gave some hope that it might be possible to remove the disease. A four-inch vertical incision was made over the outer part of the growth, the middle of the incision being about one inch below the level of the umbilicus. On turning up the great omentum a hard, irregular mass was found occupying the upper part of the ascending colon, but invading the mesocolon and, by its contraction, dragging the transverse colon and cæcum towards itself. The tumour and the adjacent parts of the intestine were delivered through the parietal wound, and isolated with sponge-cloths. One of O'Hara's forceps was placed on the ileum, and another on the transverse colon well away from the growth. My own intestinal clamps were placed on the gut, about two inches nearer the tumour in each case. The transverse colon was divided close to the proximal side of the O'Hara forceps, and the removal of the growth commenced from this point. The gastro-colic omentum, mesocolon, mesocæcum, and the portion of the mesentery attached to the lower ileum were tied off by a series of mass sutures passing through these structures. The ileum was then divided on the distal side of the other O'Hara forceps, about six inches from the ileo-cæcal valve, and the whole mass thus removed. The two O'Hara forceps were approximated so that the cut end of the ileum came in close contact with the cut end of the transverse colon, and ileo-colostomy by end-to-end union was effected by Halsted's sutures. The mesentery of the ileum was also attached to the remains of the transverse mesocolon. The parts removed consisted of six inches of ileum, the cæcum and vermiform appendix, the whole of the ascending colon with the growth, about two-thirds of the transverse colon, and the whole of the great omentum. Two lymphatic glands, the size of small marbles, were removed from the ascending mesocolon. The growth involved the whole circumference of the gut, and was about three inches in diameter. Solid food was given five days after operation; the bowels acted spontaneously on the eleventh day; and the patient began to get up at the end of three weeks after operation, his recovery being rapid and complete.

In addition to the case above recorded, I used the O'Hara forceps in Case 46. They are most useful in holding the gut in place during the process of suturing; and as they are withdrawn from the bowel, they are not open to the objections which may be urged against many of the mechanical contrivances in common use for enterorraphy.

*H.*—**Operations for Appendicitis.**—Cases 2, 3, 7, 8, 10, 11, 14, 15, 20, 21, 24, 25, 26, 34, 35, 36, 37, 41, 45 and 47.

In Case 7 the appendix was gangrenous; and in this and in Case 14 the peritonitis was of the diffuse type. Cases 35, 36 and 37 were associated with severe but localized peritonitis, and in these cases only was it thought desirable not to make any attempt to deal with the appendix itself.

The successful issue in the following case of acute perforative appendicitis was largely due to the adoption of abdomino-vaginal drainage.

Case 7.—**Gangrenous Appendicitis — Appendicectomy.**—The patient had some abdominal pain for two days, but had been out the day before I saw her. There was slight shivering; temperature 102°; and abdominal pain, more marked in the right iliac fossa, gradually increased in intensity during the night. Five and a half hours before operation the pain became agonising, in spite of opium. The legs were drawn up; the tongue was dry; the pulse was small, 120 per minute; the abdomen was distended, and there was acute tenderness in the right iliac fossa. An oblique incision was made over the appendicular region. On opening the peritoneum, about half a pint of a dirty yellow, stinking fluid escaped from the general peritoneal cavity. The coils of intestine were much congested. A few flakes of lymph existed near an agglutinated mass in the appendicular region. Involved in this mass was the appendix, four and a half inches long, almost entirely gangrenous and having a large perforation about a quarter of the way down. After throwing a ligature round its base, the appendix was removed. Stercoraceous fluid, which had escaped from the intestine, was found bathing the pelvis and the flank without any limiting adhesions. A hole was made from the vagina into the peritoneal cavity through Douglas's pouch, and a large rubber tube was passed through from the abdominal wound to the vagina. Another large tube was passed from the abdominal wound into the hollow of the sacrum; and a third tube was passed from the parietal wound through a counter-puncture in the right flank, to drain the pouch below the right kidney. The head of the bed was raised, in order that the exudation might be confined to the pelvis. The patient was extremely ill for some days; but in spite of the severity of the attack the tubes were removed in the course of three weeks, and the patient made an excellent recovery.

The baneful effect of appendicitis upon the female generative organs is perhaps not so fully recognised as its importance deserves. Salpingo-öophoritis not uncommonly ensues; and this in turn may result in tubo-ovarian abscess, or other serious mischief, necessitating a severe, difficult, and often complicated operation. I have had several such cases, and the following is a good example of its class.

Case 45.—**Appendicitis—Broad Ligament Cysts—Appendicectomy—Salpingo-oöphorectomy—Enucleation of Cysts.**—In the twelve months preceding operation the patient had two attacks of what appeared to be severe pelvic cellulitis, one of which nearly cost her her life. She was never entirely free from pain for long, and had been reduced to a condition of chronic invalidism. There was tenderness over McBurney's point; and behind the uterus, which was fixed, a matted mass could be felt in the neighbourhood of the Fallopian tubes and broad ligaments. An oblique incision was made in the right iliac fossa. A short dilated appendix, bound down by firm adhesions, was brought to the surface and removed in the usual way. On examining the pelvis, numerous and dense adhesions were felt matting the uterus (which was the size of a two months' pregnancy) and ovaries to the parietes and intestines. A median incision was made between the umbilicus and pubes, and the patient placed in the Trendelenburg posture. Both Fallopian tubes seemed bent on themselves behind the uterus. In the right broad ligament several cysts were broken into, in attempts to enucleate them, and the complexity of the adhesions made it impossible to deal with the somewhat ragged cavity which was left. After some difficulty, the left ovary, Fallopian tube, and some cysts were brought to the surface, and the mass tied off and removed. Both parietal wounds were then completely closed. The dilated appendix contained three small fæcal concretions below a sharp constriction half an inch from the cæcal end. The portion of left broad ligament removed contained three cysts, rather smaller than grapes. The patient made an uninterrupted recovery.

*J.*—**Exploratory Operations.**—Cases 4, 16, 22, 28, 30 and 40.

With the exception of Cases 22 and 30, all these operations were performed with a view to affording relief in cases of malignant disease. Case 22 had symptoms of chronic intestinal obstruction, which entirely subsided after cœliotomy; and Case 30 was one of acute pancreatitis, and has already been referred to under "Operations for diseases of the pancreas."

